# Implant‐supported removable partial dentures compared to conventional dentures: A systematic review and meta‐analysis of quality of life, patient satisfaction, and biomechanical complications

**DOI:** 10.1002/cre2.521

**Published:** 2022-01-11

**Authors:** Octave N. Bandiaky, Dohoue L. Lokossou, Assem Soueidan, Pierre Le Bars, Moctar Gueye, Elhadj B. Mbodj, Laurent Le Guéhennec

**Affiliations:** ^1^ Division of Fixed Prosthodontics University of Nantes Nantes France; ^2^ School of Dentistry University of El Hadji Ibrahima Niasse Dakar Sénégal; ^3^ Department of Periodontology, Rmes U1229 University of Nantes Nantes France; ^4^ Division of Fixed Prosthodontics CHU Nantes Nantes France; ^5^ Department of Odontology, Clinic of Fixed and Removable Prosthodontics University of Dakar Dakar Senegal; ^6^ Department of Prosthetic Dentistry, Faculty of Dentistry University of Nantes Nantes France

**Keywords:** implant‐supported removable partial dentures, patient's satisfaction, quality of life, removable partial denture

## Abstract

**Objectives:**

The purpose of this systematic review and meta‐analysis was to compare implant‐supported removable partial dentures (ISRPDs) with distal extension removable partial dentures (DERPDs) in terms of patient‐reported outcome measures (PROMs: patients' quality of life and satisfaction) and to determine mechanical and biological complications associated with ISRPDs.

**Material and Methods:**

An electronic search was performed on four databases to identify studies treating Kennedy class I or II edentulous patients and which compared ISRPDs with DERPDs in terms of PROMS and studies, which evaluated mechanical and biological complications associated ISRPDs. Two authors independently extracted data on quality of life, patient satisfaction, and biomechanical complications from these studies. The risk of bias was assessed for each study, and for PROMs, the authors performed a meta‐analysis by using a random‐effects model.

**Results:**

Thirteen articles were included based on the selection criteria. The difference in mean scores for quality of life (30.5 ± 1.8; 95% confidence interval [CI], 24.9–36.1) and patient satisfaction (−20.8 ± 0.2; 95% CI, −23.7 to −17.8) between treatments with conventional and implant‐supported removable dentures was statistically significant (*p* < .05). Implant‐supported removable dentures improved patients' overall quality of life and satisfaction. Some mechanical and biological complications, such as clasp adjustment, abutment or implant loosening, marginal bone resorption, and peri‐implant mucositis, were noted in ISRPDs during patient follow‐up. Studies assessing PROMs were very heterogeneous (*I* ^2^ = 65%, *p* = .85; *I* ^2^ = 75%, *p* = .88).

**Conclusions:**

ISRPDs significantly improved quality of life and patient satisfaction. Some mechanical and biological complications have been associated with ISRPDs treatment, requiring regular monitoring of patients to avoid the occurrence of these complications.

## 1 INTRODUCTION

Distal extension removable partial dentures (DERPDs) are a suitable treatment option that improves stomatognathic functions, which are still widely used in the rehabilitation of Kennedy‐Applegate class I or II partially edentulous patients (Gonçalves et al., 2014a; Vanzeveren et al., 2003). However, this type of prosthesis is associated with increased alveolar bone resorption, caries lesions on teeth, and psychologically less acceptable treatment (Knezović Zlatarić et al., 2002). Moreover, DERPDs present many biomechanical problems (unsatisfactory retention and stability), which may compromise masticatory efficiency (Cunha et al., 2008). Additionally, its limited functional and aesthetic properties (Shala et al., 2016) and its relatively high complication or failure rate (Knezović Zlatarić et al., 2002; Vermeulen et al., 1996; Wagner & Kern, 2000) explain why DERPDs can be a source of discomfort and dissatisfaction for patients (Armellini et al., 2008; Bilhan et al., 2012). For these reasons, some patients rehabilitated with DERPD do not wear their prostheses regularly (Vanzeveren et al., 2003), hence the need for clinicians to consider other treatment alternatives as dental implants. According to the literature, dental implants are a highly successful treatment option for the replacement of missing teeth (Albrektsson et al., 1986; Howe et al., 2019; Pjetursson et al., 2012; Weber & Sukotjo, 2007). Their long‐term survival rate was assessed in many systematic reviews (Hjalmarsson et al., 2016; Howe et al., 2019; Moraschini et al., 2015) which reported various results. The authors of these reviews concluded that this survival rate at 10‐years follow‐up was over 92.8% (95% confidence interval [CI]: 90–94.8) (Hjalmarsson et al., 2016; Howe et al., 2019; Moraschini et al., 2015). However, the presence of bone defects at the implantation site limits at times the availability of bone tissue for placing an adequate number of implants. Fortunately, there are other therapeutic solutions that make it possible to overcome this obstacle. It is notably about the use of dental implants with reduced dimensions (Threeburuth et al., 2018) or preliminary bone reconstruction of the edentulous jaw, a process that can uses the combination of bone substitutes with autologous mesenchymal stem cells or autologous bone grafting (Arinzeh et al., 2005; Finkemeier, 2002; Gjerde et al., 2017, 2018). Likewise, bone substitutes of animal, human or synthetic origin may be used alone to reconstruct small defects (Malard et al., 2007). However, these alternative treatments are often associated with increased cost, treatment time, and postoperative morbidity.

Implant‐supported removable partial dentures (ISRPDs) have been suggested as a minimally invasive approach for partially edentulous patients and are a suitable alternative to DERPDs without compromising implant success while improving the quality of life and satisfaction of patients when compared with DERPDs (De Carvalho et al., 2001; Ganz, 1991; Giffin & Dent, 1996; Kuzmanovic et al., 2004; Mijiritsky & Karas, 2004; Ohkubo et al., 2008; Uludag & Celik, 2006; Park et al., 2020). The IRSPDs provide cost‐effective treatment. This treatment option not only increases the retention of the prosthesis and hence limits lateral and vertical displacement of the removable partial denture, but it also distributes masticatory forces more effectively along the prosthesis and the adjacent teeth (Cho, 2002). It also increases patient satisfaction and improves chewing ability, phonetics, and esthetics, since sometimes the unesthetic vestibular bracing arms can be removed (Ohkubo et al., 2007; Shahmiri & Atieh, 2010). Previous studies have reported that ISRPDs are of both functional and aesthetic interest. It is a preferable treatment option for patients with complaints about their DERPDs (Mijiritsky & Karas, 2004; Uludag & Celik, 2006; Wismeijer et al., 2013). The relevant literature demonstrates that the additional retention provided by implants increases stability (Ohkubo et al., 2008) and thus improves masticatory efficacy and patient satisfaction (Cho, 2002; De Freitas et al., 2012; Goiato et al., 2018; Grossmann et al., 2009; Suzuki et al., 2017; Wismeijer et al., 2013; Zancopé et al., 2015). In addition to functional comfort, there is also an aesthetic gain through the removal of unsightly clasps in the anterior areas (Grossmann et al., 2008). ISRPDs also improve the quality of life of patients wearing appliances without the need for invasive surgery (bone grafts, sinus lift, etc.) (Cho, 2002; Goiato et al., 2018; Wismeijer et al., 2013). However, there is a lack of systematic reviews and meta‐analyses providing clear scientific evidence of the long‐term therapeutic efficacy of this prosthesis compared to conventional dentures (DERPDs). For these reasons, the purpose of this systematic review and meta‐analysis was to compare ISRPDs and DERPDs in terms of the patient‐reported outcome measures (PROMs: quality of life and patient satisfaction) and to determine the mechanical and biological complications associated with ISRPDs. The null hypothesis was that no difference would be found in the quality of life and satisfaction of patients rehabilitated with ISRPDs compared to those fitted with DERPDs.

## 2 MATERIALS AND METHODS

### 2.1 Protocol and study questions

This systematic review and meta‐analysis were conducted in accordance with the PRISMA (Preferred Reporting Items for Systematic reviews and Meta‐Analyses) recommendations (Moher et al., 2009) and the Cochrane Guidelines (Cumpston et al., 2019). The review was not registered in PROSPERO before data collection.

This study deals only with data from clinical studies published in bibliographic databases or specialized journals, its aspect does not require the approval of the ethics committee nor the “protection of human subjects and animals in research” or informed consent.

### 2.2 Type of intervention, primary and secondary outcomes

The participants comprised patients with Kennedy Class I or II edentulism; the intervention was patients rehabilitated with ISRPDs in comparison with those rehabilitated with DERPDs. The primary outcome of studies was the patient‐reported outcome measures (quality of life and patient's satisfaction) evaluated after DERPD and ISRPD treatment. A population, intervention, control, and outcome (PICO) were used to formulate a primary outcome question: Does the use of ISRPD improve quality of life and patient satisfaction than DERPD. Quality of life is a patient's judgment of various aspects of their physical, health, social and psychological well‐being. Patient satisfaction refers to the sense of well‐being that patients feel following prosthetic treatment. The secondary outcomes were the mechanical and biological complications (marginal bone loss, tooth mobility, periodontal pocket, implant survival rate) associated with ISRPDs.

### 2.3 Inclusion and exclusion criteria

The review included human clinical controlled studies evaluating patient‐reported outcome measures and biomechanical complications associated with ISRPDs, and in which patients were rehabilitated first with a DERPD and then with an ISRPD. Articles from studies with no available data, prosthetic rehabilitations other than ISRPDs and DERPDs, clinical report cases, and literature reviews were excluded from this analysis. Similarly, studies that did not compare ISRPD to DERPD in terms of patients' quality of life or satisfaction, that did not evaluate the clinical complications of ISRPDs, or with fewer than 10 participants were excluded from this analysis.

### 2.4 Search strategy and databases

Four databases (MEDLINE/PubMed, Scientific Electronic Library Online [SciELO], Cochrane Library, and ScienceDirect) were electronically searched to identify all the relevant studies for articles published up to 2021 with no date or language limitations. The search strategy at the database level remains identical for all these databases. A supplemental manual search was performed by reviewing the reference lists of the related papers. Publication and selection bias was minimized in the bibliographic search by utilizing a comprehensive search strategy that included controlled vocabulary and free terms. The following keywords combined with Boolean operators and Medical Subject Headings (MeSH), Health Sciences Descriptors (DeCS), and Embase Subject Headings (Emtree) were used in all databases ([removable partial denture OR Kennedy Class I partial edentulous OR distal‐extension removable partial denture OR jaws OR edentulous OR denture displacement OR conventional RPDs OR partial denture OR removable] AND [dental implants OR implant mechanical complications OR implant‐supported removable partial dentures OR patients satisfaction OR patients quality of life OR PROMs OR randomized controlled trials OR comparatives studies OR prospective studies OR implant survival rate OR periodontal pocket OR tooth loss OR bone loss OR implant loss]). A manual search was also performed in the following journals: Clinical Implant Dentistry and Related Research, Clinical Oral Implants Research, Journal of Dentistry, Journal of Oral Implantology, The Journal of Prosthetic Dentistry, and the Journal of Clinical and Experimental Dental Research.

### 2.5 Selection procedure and data extraction

A calibration of two reviewers (L. L and O.N.B.) was performed before the selection of studies, to determine inter‐examiner agreement in the study‐selection process for publication in the PubMed/MEDLINE, SciELO, Cochrane Library, and ScienceDirect databases and in specialized journals. This calibration was performed according to the method described by Landis and Koch (1977). After achieving an appropriate level of agreement (*κ* ≥ 0.81), the reviewers (L.L and O.N.B.) performed a methodical analysis of all studies titles, abstracts and full text, independently. Any disagreements were resolved by discussion to find a consensus during study selection and data extraction. The selection of studies at the database level was performed in four steps. First, the retrieved articles were imported into a bibliographic reference management software program (Zotero; Corporation for Digital Scholarship), where duplicates were removed. In the second step, the titles of the different references were independently reviewed by L. L and O. N. B., and articles not related to the topic were eliminated. Then, the abstracts and the full text of the study were read to apply the inclusion and exclusion criteria in the third step. At this stage, any studies not meeting the inclusion criteria were excluded, and the reasons for exclusion were recorded for each study. Data extraction and synthesis were performed by L. L. using Microsoft Excel 2010 (Excel 2010; Microsoft Corp). The information was verified and confirmed by O. N. B. The following data were collected: author and year of publication, study design, number and age of participants, implant system/diameter/length, attachment systems, Kennedy class and edentulous arch, study group, follow‐up period, marginal bone loss; and implant survival rate, variables that were assessed and the method of assessment of these variables, as well as the main results.

### 2.6 Risk of individual bias of the studies

The risks of bias were evaluated for the totality of the studies included according to a modified MINORS scale (Methodological Index for Non‐Randomized Studies) of Tsirogiannis et al. (2016). This scale consisted of 10 items, with 2 additional items proposed for in vivo studies. Each item is scored from 0 to 2; for most items, 0 indicates that the content of the item is not reported, 1 indicates that the content is reported but inadequately, and 2 indicates that it is sufficiently reported. The risk of bias could be weak, moderate, or high (Table 1).

**Table 1:** Modified methodological index assessing level risk bias in nonrandomized studies (MINORS)

Evalaution scale	Score attributed	Clinical studies										
		Jensen (2016)	Campos and Gonçalves (2014, 2015)	Gates III (2014)	Bellia (2020)	Jensen (2016)	Bortolini (2011)	Wismeijer and Payne (2013, 2017)	Mijiritsky (2013)	Grossmann (2008)	Ortiz Puigpelat (2014)	Oh (2020)
Clearly stated purpose	0: not reported, 1: reported but inadequate, 2: reported and adequate.	2	2	2	2	2	2	2	2	2	2	2
Study design	0: not reported, 1: reported but inadequate, 2: reported and adequate.	2	2	2	2	2	1	2	2	2	2	2
Randomization	0: not reported, 1: reported but inadequate, 2: reported and adequate.	1	0	0	0	1	0	0	0	0	0	0
Formation and comparability of groups	0: not reported, 1: reported but inadequate, 2: reported and adequate.	2	2	2	2	2	0	2	0	0	0	0
Characteristics of the study	0: not reported, 1: reported but inadequate, 2: reported and adequate.	2	2	2	2	2	2	2	0	2	2	2
Factor(s) studied, or parameters measured, are they well described?	0: not reported, 1: reported but inadequate, 2: reported and adequate.	2	2	2	2	2	2	2	2	2	2	2
Primary endpoint	0: not reported, 1: reported but inadequate, 2: reported and adequate.	2	2	2	2	2	2	2	2	2	2	2
Appropriate sample size	0: not reported, 1: reported but inadequate, 2: reported and adequate.	2	1	2	1	2	2	1	2	2	1	2
Statistical power and justification of the number of participants	0: not reported, 1: reported but inadequate, 2: reported and adequate.	2	2	2	2	2	0	0	0	0	0	1
Statistical analysis	0: not reported, 1: reported but inadequate, 2: reported and adequate.	2	2	2	2	2	0	2	0	2	2	2
Prospective data collection	0: not reported, 1: reported but inadequate, 2: reported and adequate.	2	2	2	1	2	2	2	2	1	1	1
Follow‐up period adapted to the objective of the study	0: not reported, 1: reported but inadequate, 2: reported and adequate.	2	2	2	1	2	2	2	2	2	2	2
	Total score	23	21	22	19	23	15	19	14	17	16	18

Abbreviation: MINORS, methodological index for nonrandomized studies.

### 2.7 Synthesis of results

Data from the various studies were extracted, and the results were synthesized. For studies in which the authors reported results as medians and interquartile ranges, the values were converted to means and SDs using the formula (q1 + median + q3)/3, where q1 indicates the 25th percentile and q3 the 75th percentile, as proposed in the study by Nagarkar et al. (2018). An approximation of the SD was obtained by applying this formula: (q3 − q1)/1.35. When several data points were reported by the authors, the most negative ones were used for the quantitative synthesis. The same was true for patient follow‐up, where the data from the longest follow‐up were retained. Meta‐analysis was performed by using R Commander™ software, and a random‐effects model (Gonçalves et al., 2014a; Higgins & Thompson, 2002). The choice of this model was justified by the fact that most of the studies were small (number of patients <30) and that the effect of the intervention measured (quality of life and patient satisfaction) was different for each of these studies, given their heterogeneity. Thus, a random‐effects model will give more weight to these small studies. When studies used the same type of intervention and comparison groups with the same outcome measure, the results were pooled with mean differences for continuous outcomes and risk ratios for dichotomous outcomes and calculated 95% CIs and *p* values for each outcome (DerSimonian and Laird method) (DerSimonian & Laird, 1986). Heterogeneity between the studies was assessed by using the Higgins *I* ^2^ statistic (Higgins & Thompson, 2002). We considered an *I* ^2^ value of 50% or more to indicate substantial heterogeneity. A sensitivity analysis based on the risk of bias of the included studies (low risk of bias vs. high or unclear risk of bias) was conducted.

## 3 RESULTS

**Figure 1:**
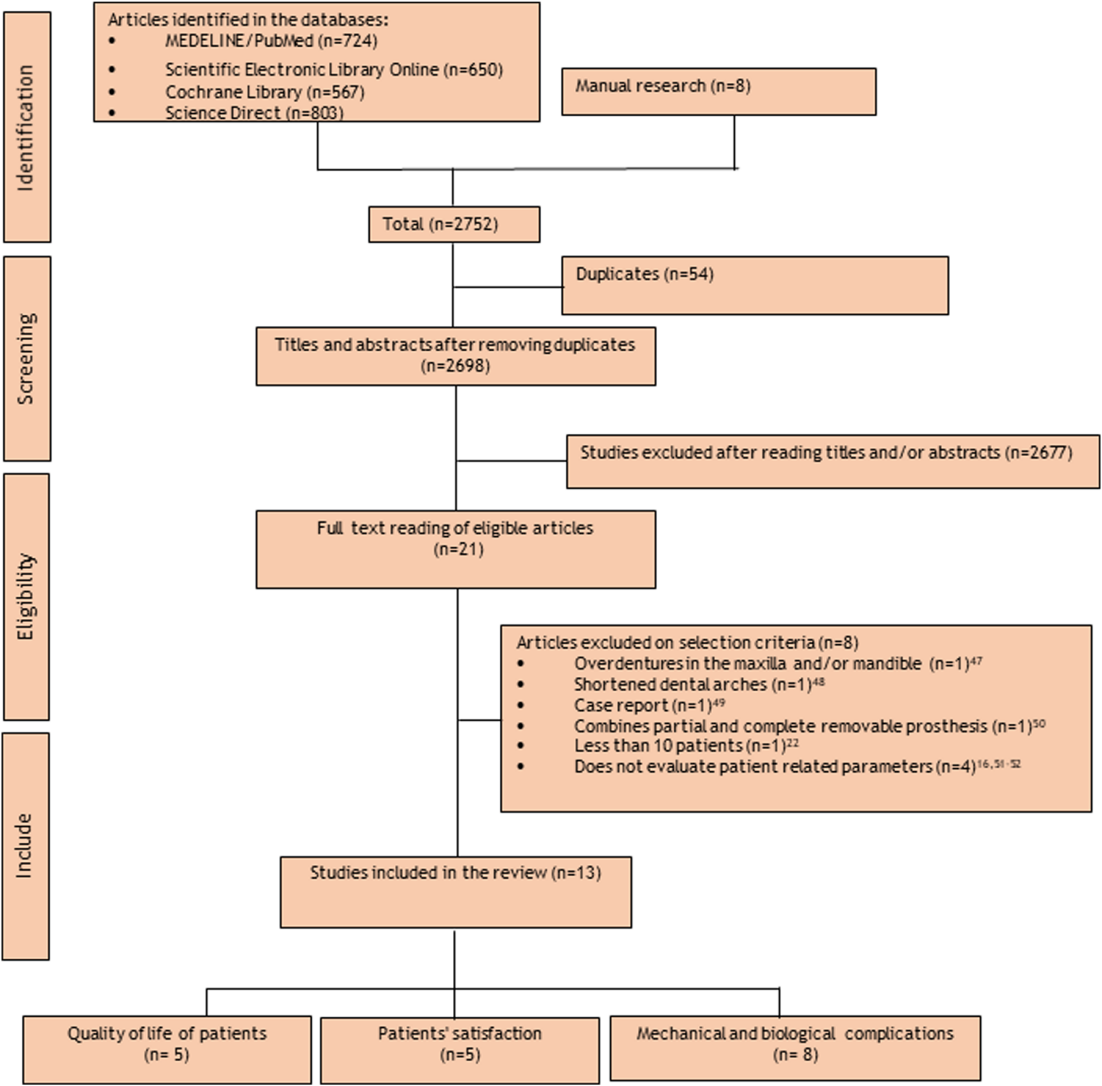
Flowchart of included studies

A bibliographic search of the four electronic databases and specialized journals identified 2752 relevant articles. After removing duplicates and title and abstract screening, 2731 articles were excluded, and 21 studies were eligible for full‐text analysis, of which eight (Bural et al., 2016; Kaufmann et al., 2009; Maeda et al., 2005; Minoretti et al., 2009; Ohkubo et al., 2008; Ohyama et al., 2020; Pellizzer et al., 2010; Threeburuth et al., 2018) studies were excluded and the reasons of their exclusion are presented in Figure 1. Thirteen studies (Bellia et al., 2020; Bortolini et al., 2011; Campos et al., 2015; Gates et al., 2014; Gonçalves et al., 2014b; Grossmann et al., 2008; Jensen et al., 2016, 2017; Mijiritsky et al., 2013; Oh et al., 2021; Ortiz‐Puigpelat et al., 2014; Payne et al., 2017; Wismeijer et al., 2013) were included in the systematic review.

### 3.1 Study characteristics

One study was RCT (Bellia et al., 2020; Campos et al., 2015; Gates et al., 2014; Gonçalves et al., 2014b; Jensen et al., 2016; Mijiritsky et al., 2013; Payne et al., 2017; Wagner & Kern, 2000; Wismeijer et al., 2013) and five (Bortolini et al., 2011; Grossmann et al., 2008; Jensen et al., 2017; Oh et al., 2021; Ortiz‐Puigpelat et al., 2014) had a prospective and retrospective design respectively. Some of these studies were conducted by the same authors on the same participants but with different outcomes (Campos et al., 2015; Gonçalves et al., 2014b; Jensen et al., 2016; Jensen et al., 2017; Payne et al., 2017; Wismeijer et al., 2013). The characteristics of these studies are described in Table 2 and the details of the search strategy are presented in a flow chart (Figure 1). A total of 238 participants with a mean age of 60.8 ± 8.01 years (range: 44.2–75.4 years) were evaluated and 535 implants were used as abutments for ISRPDs (500 conventional dental implants and 35 mini‐dental implants). The follow‐up duration of the study varied from 2 to 180 months. The implant diameters ranged from 3.3 to 6 mm and the length ranged from 6 to 15 mm. The most used implant system was Straumann, followed by Neodent, Zimmer Dental Implant, and Biomet 3i. The most of participants were partially dentate mandibles characterized by many missing teeth. Kennedy Class I was the most dominant and the ball attachment system was used in most studies (Bortolini et al., 2011; Campos et al., 2015; Gates et al., 2014; Gonçalves et al., 2014b; Grossmann et al., 2008; Jensen et al., 2017; Mijiritsky et al., 2013; Payne et al., 2017; Wismeijer et al., 2013). Some studies compared ISRPDs to DERPDs in terms of the PROMs (quality of life and patient satisfaction) (Bortolini et al., 2011; Campos et al., 2015; Gates et al., 2014; Gonçalves et al., 2014b; Grossmann et al., 2008; Jensen et al., 2016; Jensen et al., 2017; Mijiritsky et al., 2013; Ortiz‐Puigpelat et al., 2014; Wismeijer et al., 2013) while others evaluated both these patients reported outcome measures and the mechanical and biological complications associated with ISRPDs (Table 2).

**Table 2:** Extracted data of included articles

Study reference	Study aim	Patients, *n* (mean age years)	Study design	Restored Arch (KA class; No. RNT	Implant, *n*	Study group (implant location)	Implant system/diameter/length	Attachment system	Variables	Mains results
Campos et al. (2015) Brazil	Evaluate OHRQOL in partially dentate subjects, comparing the use of DERPD and ISRPD	12 (62.6)	Prospective comparative study	Mn (I; 6)	24	Group 1 (*n *= 12): DERPD Group 2 (*n *= 12): DERDP + 2 implants (36–46)	Titamax‐Neodent/3.75–6 mm/7–13 mm	Ball (O'ring; Neodent)	OHRQOL	The strategic placement of osteointegrated implants in the posterior region of patients presenting mandibular Kennedy Class I edentulism improved significantly their OHRQOL
Gates III et al. (2014) USA	Evaluate OHRQOL for patients treated with DERPDs compared to ISRPDs	17 (61.5)	Prospective study	Mn (I, II; 4–11)	30	Group 1 (*n *= 17): DERPD + 1 or 2 implant(s) submerged (36 or 36–46) Group 2 (*n *= 17): DERPD + 1 or 2 implant(s) (36 or 36–46)	AstraTech AB/4 mm/6 mm	Ball	OHRQOL Mechanical and biological complications	ISRPDs substantially improved the OHQoL in patients with mandibular Kennedy Class I and Kennedy Class II partial edentulism. The use of short implants (e.g., 4.0 9 6 mm implants) may be considered to support ISRPD, but with caution due to inadequate long‐term follow‐up
Ortiz‐Puigpelat et al. (2014) Spain	Report on the clinical performance of ISRPD with Locator abutments in different partial edentulism situations, with a mean follow‐up period of 28.6 months	12 (75.4)	Retrospective case series	Mx, Mn (I, II; 4–7)	24	Partially edentulous patients	Screwplant (Implant Direct)/3.7–4.7 mm/8–13 mm	Locator	Patient satisfaction Mechanical and biological complications	Treatment with ISRPD can improve the patient's function, phonetics, and esthetics without the need for extensive bone regeneration surgeries and prosthodontic rehabilitations
Gonçalves et al. (2014b) Brazil	Evaluate patient satisfaction after use of DERPD and ISRPD	12 (62.6)	Prospective comparative study	Mn (I; 6)	24	Group 1 (*n *= 12): DERPD Group 2 (*n *= 12): DERPD + 2 implants (36*–*46)	Titamax‐Neodent/3.75–6 mm/7–13 mm	Ball (O'ring; Neodent)	Patient satisfaction Mechanical and biological complications	Implant‐retained and‐supported removable prostheses improve retention and stability, minimize rotational movements, and significantly increase participant satisfaction
Jensen et al. (2016) The Netherlands	Assess the benefits of implant support to DERPD in partially dentate patients to determine the most favorable implant position	30 (60.9)	Cross‐over RTC	Mn (I; 6)	120	Group 1 (*n *= 30): DERPD Group 2 (*n *= 30): DERPD + 2 implants (PM) + 2 implants (M)	Straumann RN/3.30–4.1 mm/6–8 mm	Locator (Zest Anchors, Inc., Escondido, California, USA)	OHRQOL	Mandibular implant support favorably influences oral health related patient‐based outcome measures in patients with a bilateral free‐ending situation. The majority of patients prefer the implant support to be in the molar region
Wismeijer et al. (2013) New zeland, Colombia and the Netherlands	Compare the levels of patient satisfaction with either DERPD and ISRPD	48 (61.7)	Multicentre prospective study	Mn (I; 6–8)	72	Control group (*n *= 12): DERPD Test groups: Groups 1, 2, 3 (*n *= 36): DERPD + 2 implants (37–47)	Straumann/4.1 mm/6–8 mm	Ball	OHRQOL Patient satisfaction	Mandibular implant‐assisted removable partial dentures are a preferable treatment option for patients with complaints about their conventional distal extension partial dentures. ISRPDs showed significant improvement on the OHIP and OHIQ scores compared to DERPDs
Jensen et al. (2017) The Netherlands	Assess performance, together with biological and technical complications, of ISRPD in mandibular Kennedy class I situations with implants placed in the anterior or posterior position	23 (59)	Retrospective study	Mn (I; 6–8)	46	Group 1: DERPD + 2 implants (PM)Group 2: DERPD + 2 implants (M)	Straumann RN/3.30–4.1 mm/6–8 mm	Ball/Locator/healingabutment	Mechanical and biological complications Patient satisfaction OHRQOL	ISRPD is a viable treatment option with a high implant survival rate and satisfied patients after a maximum of 16 years. Technical and biological complications should be anticipated. Anteriorly placed implants performed slightly better
Bortolini et al. (2011) Italy	Evaluate the long‐term outcomes of removable partial dentures (RPDs)retained (but not supported) by dental implants	32 (56.8)	Retrospective Study	Mn (I, II, III)	64	Group 1 (*n *= 19): Class I + ISRPDs Group 2 (*n *= 10): Class II + ISRPDs Group 3 (*n *= 3): Class III + ISRPDs	Branemark MKIII‐Nobelpharma/3.75–5 mm/10–15 mm	Ball	Patient satisfaction Mechanical and biological complications	Implant‐retained RPDs are a reliable intermediate solution that can reduce biological and economic costs while maintaining implant treatment benefits and the ease of RPD procedures. Periimplant soft tissues and residual edentulous ridges remain stable over time
Mijiritsky et al. (2013) Russia	Describe the long‐term follow‐up of cases treated with ISRPD after at least 15 years.	20 (56)	Prospective study	Mx and Mn (I, II; 6–8)	42	Partially edentulous patients	Zimmer Dental; Friadent; MIS Implants/3.7–5 mm/10–13 mm	Ball	Patient satisfaction Mechanical and biological complications	No implant failure was noted during follow‐up, resulting in a rate for implant survival of 100% for the study. Marginal bone loss around implants and prosthetic complications were minor and included one rest rupture. All patients were satisfied and reported good chewing ability and stability of the prosthetic devices
Grossmann et al. (2008) Israel	Evaluate the survival of endosseous dental implants used in restoring partially edentulous patients with ISRPD	23 (44.2)	Retrospective case series	Mx, Mn (NR)	44	Partially edentulous patients	Straumann/4.1 mm/6–10 mm	Ball	Patient satisfaction Biological complications	ISRPD could serve as a longterm predictable treatment modality. Careful patient selection, with an appropriate maintenance and recall system, is recommended to obtain satisfactory results
Oh et al. (2021) Republic of Korea	Evaluate the clinical status and complications of IARPDs combined with implant surveyed prostheses	24 (67.4)	Retrospective clinical study	MnMx (I, II, IV; 2–5)	80	Partially edentulous patients treated with an IARPD	NR	NR	Mechanical and biological	IARPDs combined with implant surveyed prostheses could be a treatment option when additional retention, support, and stability are required for partial edentulism
Payne et al. (2017) New zeland, Colombia and the Netherlands	To determine implant survival and prosthodontic maintenance of implant‐assisted mandibular removable partial dentures	48 (61.7)	Multicentre prospective study	Mn (I; 6–8)	72	Control group (*n*= 12): DERPD Test groups: Groups 1, 2, 3 (*n *= 36): DERPD + 2 implants (37–47)	SLA active; RN, Straumann/4.1 mm/6–10 mm	Ball	Biological complications	Late implant failures and increased prosthodontic maintenance when an attachment system is used identify the need for further research, including more robust statistical analyses
Bellia et al. (2020)	Evaluate the survival at 1 and 4 years of short implants retaining removable partial dentures (RPDs) in Kennedy Class I and II edentulism	20 (61.5)	Prospective study	Mx, Mn (I, II; NR)	35	Partially edentulous patients	Super Short 3i Implantes (NanoTiteSurface)‐Biomet 3i/5–6 mm/5–6mm	Locator	Biological complications	The use of short implants for retaining RPDs may be considered a viable treatment option for patients with distal edentulism and contraindications for more complex implant rehabilitation

Abbreviations: DERPD, distal extension removable partial denture; IARPD, implant‐assisted removable partial dentures; ISRPD, implant‐supported removable partial denture; KA, Kennedy‐Applegate; M, molar; Mn, mandible; Mx, maxilla; No, number; NR, not reported; OHRQOL, oral health‐related quality of life; PM, premolar; RCT; randomized controlled trial; RNT, remaining natural teeth; RPDs, removable partial dentures.

### 3.2 Primary outcome of the studies (PROMs: quality of life and patient's satisfaction)

**Figure 2:**
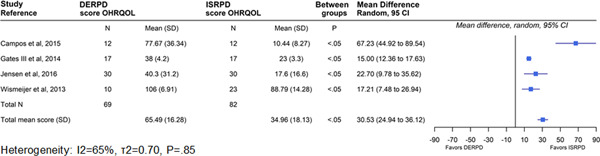
Mean score OHIP questionnaires between 2 treatment modalities (DERPD vs. ISRPD). Wilcoxon signed‐rank test. Significant at *p* < .05. CI, confidence intervals; DERPD, distal removable partial denture; ISRPD, implant‐supported removable partial dentures; OHIP, oral health impact profile; OHRQOL, oral health‐related quality of life; SD, standard deviation

**Figure 3:**
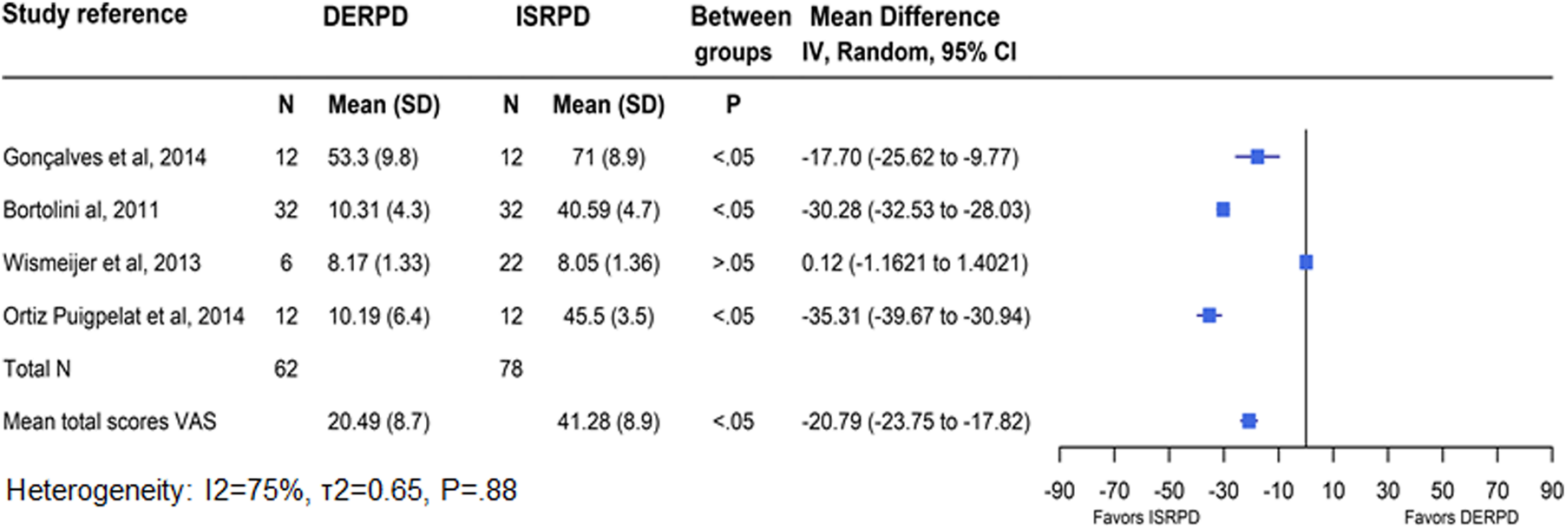
Mean VAS score assessing participant's satisfaction. Significant at *p* < .05. DERPD, distal removable partial denture; ISRPD, implant‐supported removable partial dentures; SD, standard deviation; VAS, visual analog scale

Concerning the primary outcome measures, some studies evaluated only or both the quality of life of patients and their degree of satisfaction after they received rehabilitation treatment with DERPDs and ISRPDs (Table 2). The quality of life and satisfaction of these patients were evaluated by using the oral health‐related quality of life questionnaire (OHRQoL), the oral health impact profile (OHIP‐49), the short‐form health survey (SF‐36), a visual analog scale (VAS), and a patient satisfaction questionnaire. All selected studies reported a significant improvement in PROMs with the use of ISRPDs as compared with DERPDs and in the investigation of Jensen et al. (2016) the patients preferred implants positioned in the molar region (50%) compared with the premolar region (30%). The instrument measures of quality of life are widely described in the literature (Gates et al., 2014; Jensen et al., 2017), and its highest score corresponds to a low level of quality of life associated with prosthetic rehabilitation. To draw relevant conclusions between the two prosthetic therapy modalities, the data from only four studies (Campos et al., 2015; Gates et al., 2014; Jensen et al., 2016; Wismeijer et al., 2013) are summarized in Figure 2, and their pooling shows a statistically significant difference in the mean quality of life score between DERPDs (65.5 ± 16.3) and ISRPDs (30.9 ± 18.1) (*p* < .05). The mean difference was (30.5 ± 1.8; 95% CI, 24.9–36.1). Due to the missing data on patients' quality of life before implant placement, the study by Jensen et al. (2017) was excluded from the quantitative synthesis of results. The studies, which assessed this parameter, showed substantial heterogeneity (*I* ^2^ = 65%, *τ* ^2^ = 0.70, *p* = .85). Patients satisfaction was evaluated in five clinical comparative studies using a VAS (Gonçalves et al., 2014b; Jensen et al., 2017; Wismeijer et al., 2013) with a numerical slider scaled from “0 = not at all satisfied” to “100 = total satisfaction” and a questionnaire (Bortolini et al., 2011; Ortiz‐Puigpelat et al., 2014) with a score between 1 and 5. Data synthesis on four studies (Bortolini et al., 2011; Gonçalves et al., 2014b; Ortiz‐Puigpelat et al., 2014; Wismeijer et al., 2013) showed that the mean values of the satisfaction scores obtained were higher in patients rehabilitated with ISRPDs (41.3 ± 8.9) than with DERPDs (20.5 ± 8.7), and the means difference (−20.79; 95% CI, −23.75 to −17.82) between the two treatment modalities was statistically significant (*p* < .05) (Figure 3). The study by Jensen et al. (2017) was also excluded from quantitative synthesis due to the missing data on patients' satisfaction at baseline. The study in which the investigators assessed the patient satisfaction were heterogeneous (*I* ^2^ = 75%, *τ* ^2^ = 0.65, *p* = .88).

### 3.3 Secondary outcomes of the studies

Ten studies included in the systematic review evaluated the mechanical and biological complications associated with ISRPDs and reported various results regarding implant survival rates, marginal bone loss around implants, abutment loosening or mobility, implant mobility, bleeding on probing or deep probing depth. They also reported the prosthetic complications such as fracture of the denture base, rest, clasps or resin. Some authors (Gonçalves et al., 2014b; Mijiritsky et al., 2013; Oh et al., 2021) reported an implant survival rate of 100% after the follow‐up period ranging from 2 to 180 months, while for others (Bellia et al., 2020; Bortolini et al., 2011; Gates et al., 2014; Grossmann et al., 2008; Jensen et al., 2017; Ortiz‐Puigpelat et al., 2014; Payne et al., 2017) this survival rate was 91.6%–97% (Table 3). The number of implants that failed was ranging from 1 to 6. The mean marginal bone loss around implants ranged between 0.64 and 2.11 mm and the mean deep pockets varied from 2 to 4 mm. Some authors (Bortolini et al., 2011; Gates et al., 2014; Grossmann et al., 2008) reported implant bleeding on probing, mobility or deep probing depth, abutment loosening, loose healing cap (Payne et al., 2017) or ball attachments replacement. Bortolini et al. (2011) reported that the peri‐implant soft tissues and marginal gingiva of most patients were slightly inflamed. In the study of Gates III et al. (2014), prosthetic complications involved clasp, fracture of denture tooth, reline of the denture base, and reprocess of DERPDs were primarily minor and could be managed within a single clinical visit. Gonçalves et al. (2014b) found stable periodontal conditions around the implants, no intrusions or mobility of teeth, and no radiographic changes in bone level after 2 months follow‐up. For their part, Jensen et al. (2017) reported that posterior implants demonstrated significantly more complications than anterior implants (peri‐implant mucositis). In their study, Bellia et al. (2020) concluded that the use of short implants for retaining DERPDs may be considered a viable treatment option for patients with distal edentulism and contraindications for more complex implant rehabilitation.

**Table 3:** Biomechanical complications associated with ISRPD

Study reference	Follow‐up mean time	Number of implants placed	Prosthetic complications and maintenance	Number of implants loss	Implant survival rate (%)
Gates III et al. (2014)	2 years	30	Clasp adjustment Fracture of denture tooth Reline of denture base Reprocess of DERPD Loss of abutment tooth Attachment replacement	1	97
Gonçalves et al. (2014b)	2 months	48	None	0	100
Bortolini et al. (2011)	8 years	64	Abutment loosening or mobility Tooth substitution Relining	4	93.7
Mijiritsky et al. (2013)	15 years	42	Marginal bone loss around implants ranged between 0 and 2 mm (mean 0.64 ± 0.6 mm) Rest rupture	0	100
Grossmann et al. (2008)	31.5 months	44	Loss of abutment tooth	2	95.5
Ortiz Puigpelat et al. (2014)	28.6 months.	24	Mobility of the metal retentive cap Fracture of framework Denture teeth wear Addition denture teeth Plastic retentive male change	2	91.6
Oh et al. (2021)	27.6 months	80	Mean marginal bone resorption of implants at 1 year after loading (0.77 ± 0.63 mm) Mean probing depth (3.4 ± 0.1 mm) Two clasp fractures, 1 rest fracture, decementation, and 1 fracture of porcelain on an implant surveyed prosthesis	0	100
Jensen et al. (2017)	8 years	46	Mean peri‐implant bone loss was 1.06 ± 0.59 in PM and 1.10 ± 0.53 Posterior implants demonstrated significantly more complications than anterior implants (peri‐implant mucositis) Loss of 3 implants in the posterior groupProbing depth (3.3 ± 1.4)	3	91.7
Bellia et al. (2020)	4 years		Bleeding on probing Deep probing depth (2‐4 mm) Implant mobility Mean bone loss was 1.04 ± 1.88 mm	2	94.3
Payne et al. (2017)	10 years		Marginal bone loss (2.11 ± 0.76) Clasp adjustments Loose healing cap Fractured wrought wire clasps on distal abutment tooth, puncture fractures of resin	6	92

Abbreviation: ISRPD, implant‐supported removable partial dentures.

As shown in Table 1, the level of risk of bias was moderate overall for all studies. The studies were of low methodological quality because half of the studies were retrospective case series.

## 4 DISCUSSION

ISRPDs can be considered as an alternative to DERPDs and implant‐supported fixed partial prostheses when placement of an adequate number of implants is limited by bone height and thickness or by financial reasons. In this situation, a small number of conventional or mini‐implants can be placed to retain and stabilize the DERPDs, provide comfort, and increase patient masticator efficacy (De Freitas et al., 2012). The objective of this systematic review and meta‐analysis was to compare ISRPDs and DERPDs in terms of PROMs (quality of life and patient satisfaction) and to determine the mechanical and biological complications associated with ISRPDs. The null hypothesis—that no difference would be found in the quality of life and satisfaction of patients rehabilitated with ISRPDs compared to those treated with DERPDs—was rejected. Meta‐analyses performed at studies that evaluated these parameters demonstrated a significant improvement in quality of life and patient satisfaction for ISRPDs compared with DERPDs. Therefore, ISRPDs can be considered a favorable treatment option improving the biomechanical behavior of the prosthesis, and the stomatognathic functions of the patients, and their quality of life or satisfaction. Our results corroborate those of Lemos et al. (2021) that reported a systematic increase in PROMs following the implant's association to DERPDs. These results may be explained by the fact that the strategic placement of implants in the posterior region under an existing removable partial prosthesis transforms Kennedy class I or II edentulism into class III edentulism improving thus the retention and stability of this prosthesis. All these advantages may be felt by the patient, explaining the substantial improvements in the quality of life and satisfaction scores observed after the placement of osseointegrated implants in a mandibular posterior region (Campos et al., 2015). In this review, patients included in the studies that evaluated the PROMs were first rehabilitated with DERPDs which were converted to ISRPDs following placement of implants in the premolar or molar region. The loading of these implants as well as the insertion of the attachment systems were carried out at least 3 months later, which was sufficient time to achieve osteointegration. In their study, Ortiz‐Puigpelat et al. (2014) reported that the treatment of partially edentulous patients with ISRPDs improves the PROMs without the need for extensive bone regeneration surgeries and prosthodontic rehabilitation. ISRPDs improve also prosthesis performance, overall patient satisfaction with respect to retention, comfort, and masticatory capacity (Gonçalves et al., 2014b). Chikunov et al. (2008) reported other advantages related to the ISRPDs: a smaller number of implants, lower cost, fewer time‐consuming clinical and laboratory procedures, simplified hygiene when compared with fixed dental prostheses, better distribution of the masticatory loads to the abutment teeth and implants, preservation of residual bone around the implants and remaining teeth, better comfort because of minimal rotational movement, treatment compliance, and possible later conversion into a complete overdenture. Most of the implants placed in the patients included in these studies were conventional types. However, Bellia et al. (2020) reported that the use of short implants for retaining DERPDs may be considered a viable treatment option for patients with distal edentulism and contraindications for more complex implant rehabilitation. The conversion of the already well‐accepted and patient‐integrated DERPDs into an ISRPDs brings more comfort during wear by limiting the prosthesis' dislocation from its supporting surfaces, particularly during mastication. Indeed, these DERPDs are known to be more vulnerable to lifting forces (Wismeijer et al., 2013). This is probably one of the main reasons why patients resort to implants to obtain a more stable and retentive prosthesis limiting food accumulation underneath the distal extension bases of the removable partial denture and decreasing the pressure on the resilient mucosa. In addition, less relining of the intaglio surface is required with implant support but hygiene maintenance of the natural teeth and implant attachment systems will be required. Therefore, our results should be interpreted with caution, our review included both prospective and retrospective studies for the evaluation of PROMs. This mix of design studies constitutes a bias in the interpretation of the results. In addition, the instruments (OHRQoL, OHIP‐49, SF‐36, VAS, and questionnaires) used to evaluate these PROMS differ from one study to another. This shows that these studies are highly heterogeneous even if the participants are their own control. In addition, the characteristics of the participants were different, some of whom were already unable to wear their DERPDs, which constitutes a selection bias.

For implant survival rate, our results were consistent with those of previous systematic reviews (De Freitas et al., 2012; Lemos et al., 2021; Park et al., 2020; Zancopé et al., 2015) which reported a low proportion of implant failure rates over a follow‐up period ranging from 6 to 180 months. Our results can be explained by the fact that most of the implants used in the selected studies were of conventional length and diameter. Indeed, it has been described in the literature that these types of implants had a better survival rate than mini‐implants (Lemos et al., 2016; Papaspyridakos et al., 2018). However, Threeburuth et al. (2018) found no difference in terms of implant survival rate between conventional‐size and mini dental implants 12 months after surgery. Some authors concluded that the mini dental implants can be applied for retaining mandibular Kennedy class I removable partial dentures in patients with little bone availability with ovedentures (Jawad & Clarke, 2019; Lemos et al., 2017; Threeburuth et al., 2018). On the other hand, some authors reported that the placement of implants at the mandibular arch may contribute to higher survival of the implants because the bone density and the thickness of the compact bone are higher in the mandible, which leads to a higher probability of survival than the maxillary arch (Lemos et al., 2017). Biological complications such as marginal bone loss around implants and pocket depth have been reported in studies that evaluated these parameters. The average marginal bone loss varies from 0.64 to 2 mm. These results corroborate those of Lemos et al. (2021) who reported in their systematic review a mean bone loss of 1.10 mm for ISRPDs, which was an acceptable mean value. Mijiritsky et al. (2013) reported a marginal bone loss around implants ranging between 0 and 2 mm (mean, 0.64 ± 0.6 mm) after 15 years of follow‐up. This marginal bone loss was >2 mm in Payne et al study after 10 years follow‐up period. In the study by Jensen et al. (2017), the mean peri‐implant bone loss was 1.06 ± 0.59 and 1.10 ± 0.53 in the premolar and molar regions respectively. Posterior implants demonstrated significantly more complications than anterior implants (peri‐implant mucositis). These results on bone loss were similar to those of Bellia et al. (2020). The average pocket depth varies from one study to another. Other biological (abutment loosening, bleeding on probing), and mechanical complications described in Table 3 have been reported by the authors of the different studies. All of these results demonstrated that the ISRPDs did not compromise the longevity of dental implants (Lemos et al., 2021), but careful planning is crucial to ensure success and prevent or minimize future problems, such as periodontal and peri‐implant bone changes. The studies included for the evaluation of these parameters were very heterogeneous due to differences in patient characteristics (age, gender, number of residual teeth, occlusal pattern, duration of follow‐up, the position of, and size of implants.

Our work has limitations, and its results should be interpreted with caution because of the low methodological quality of the included studies, the small number of participants, and the short follow‐up period for some studies. These are mainly retrospective studies with a low level of scientific evidence. There is a lack of randomized controlled studies dividing patients into parallel groups and evaluating their quality of life and level of satisfaction and the biomechanical complications associated with each type of prosthetic rehabilitation. However, some positive points emerge from this study, and the patients served as their own controls, which limits the interindividual variability of the results.

## 5 CONCLUSIONS

A qualitative and quantitative synthesis of the data reported in the included studies indicates that:

1. ISRPDs significantly improved patients' quality of life and satisfaction compared to DERPDs.
2. Some mechanical and biological complications were observed following the completion of the ISRPDs.
3. Longitudinal prospective clinical studies in a large population are needed to confirm the stability of the results related to the quality of life and patient satisfaction and to evaluate the biomechanical complications associated with ISRPDs.

## CONFLICT OF INTEREST

The author declares that there is no conflict of interest.

## AUTHOR CONTRIBUTIONS

*Conducting the literature review, selecting articles, supervision, conception and design of the literature review, collecting and synthesizing data, evaluation of the level of risk of bias, drafting the article and writing of the statistical analysis part, writing the manuscript and final approval of the version to be submitted*: Octave N. Bandiaky. *Conducting the literature review and the level of risk of bias, selecting articles, collecting and synthesizing data, and final approval of the version to be submitted*: Dohoue L. Lokossou. *Analysis and interpretation of data, proofreading and correction of the manuscript before submission for publication and final approval of the version to be submitted*: Assem Soueidan, Pierre Le Bars, Moctar Gueye, Elhadj B. Mbodj, and Laurent Le Guéhennec.

## Data Availability

The datasets collected and/or analyzed during the current systematic review and meta‐analysis are available from the corresponding author on reasonable request.
